# Predicting compositions of microbial communities from stoichiometric models with applications for the biogas process

**DOI:** 10.1186/s13068-016-0429-x

**Published:** 2016-01-22

**Authors:** Sabine Koch, Dirk Benndorf, Karen Fronk, Udo Reichl, Steffen Klamt

**Affiliations:** Max Planck Institute for Dynamics of Complex Technical Systems, Sandtorstr. 1, 39106 Magdeburg, Germany; Otto-von-Guericke-University, Universitätsplatz 2, 39106 Magdeburg, Germany; Harz University of Applied Sciences, Friedrichstrasse 57-59, 38855 Wernigerode, Germany

**Keywords:** Microbial communities, Anaerobic digestion, Hierarchical optimization, Prediction of community composition, Stoichiometric and constraint-based modeling

## Abstract

**Background:**

Microbial communities are ubiquitous in nature and play a major role in ecology, medicine, and various industrial processes. In this study, we used stoichiometric metabolic modeling to investigate a community of three species, *Desulfovibrio vulgaris*, *Methanococcus maripaludis*, and *Methanosarcina barkeri*, which are involved in acetogenesis and methanogenesis in anaerobic digestion for biogas production.

**Results:**

We first constructed and validated stoichiometric models of the core metabolism of the three species which were then assembled to community models. The community was simulated by applying the previously described concept of balanced growth demanding that all organisms of the community grow with equal specific growth rate. For predicting community compositions, we propose a novel hierarchical optimization approach: first, similar to other studies, a maximization of the specific community growth rate is performed which, however, often leads to a wide range of optimal community compositions. In a secondary optimization, we therefore also demand that all organisms must grow with maximum biomass yield (optimal substrate usage) reducing the range of predicted optimal community compositions. Simulating two-species as well as three-species communities of the three representative organisms, we gained several important insights. First, using our new optimization approach we obtained predictions on optimal community compositions for different substrates which agree well with measured data. Second, we found that the ATP maintenance coefficient influences significantly the predicted community composition, especially for small growth rates. Third, we observed that maximum methane production rates are reached under high-specific community growth rates and if at least one of the organisms converts its substrate(s) with suboptimal biomass yield. On the other hand, the maximum methane yield is obtained at low community growth rates and, again, when one of the organisms converts its substrates suboptimally and thus wastes energy. Finally, simulations in the three-species community clarify exchangeability and essentiality of the methanogens in case of alternative substrate usage and competition scenarios.

**Conclusions:**

In summary, our study presents new methods for stoichiometric modeling of microbial communities in general and provides valuable insights in interdependencies of bacterial species involved in the biogas process.

**Electronic supplementary material:**

The online version of this article (doi:10.1186/s13068-016-0429-x) contains supplementary material, which is available to authorized users.

## Background

Microbial communities are ubiquitous in nature and play a major role in ecology, medicine, and some industrial processes. They are involved in biogeochemical cycles [1–3] and the human microbiome seems to be of high relevance for human health [4, 5]. An example for a biotechnological application involving a complex microbial community is anaerobic digestion for biogas production which will be the focus of this study.

In a microbial community, various species interact closely with each other. Each species has different requirements for growth and several factors like temperature, pH value, and nutrient supply can thus influence the community structure. Consequently, microbial communities are very complex systems and mathematical modeling has been shown to be a valuable tool to gain better understanding about the relevant interactions and community behavior [6]. In biogas plants, for example, process failures (e.g., acidification) may occur if the microorganisms involved are not in a stable steady state [7]. Here, modeling might help to identify reasons for process failures and to predict optimal conditions for a stable process.

In recent years, different strategies for modeling have been developed to investigate factors that shape microbial communities and to predict relevant interactions under different growth conditions. One of those methods is stoichiometric and constraint-based metabolic modeling that has been successfully applied for analyzing genome-scale metabolic networks of single-species [8–10] and was extended to the community level in recent years [11–15]. Stolyar et al. [11] were the first to create a two-species stoichiometric model consisting of *Desulfovibrio vulgaris* and *Methanococcus maripaludis* to analyze key characteristics of the community including its composition and metabolite exchange fluxes. Metabolic models have also been used to predict interactions (cooperation and competition) in different media [16] and emergent biosynthetic capacities for different pairs of species [17]. Taffs et al. [18] created a model which contained three different functional guilds and used elementary modes to analyze mass and energy fluxes in a microbial community. Finally, stoichiometric metabolic models have also been analyzed with dynamic flux balance analysis (FBA) [15, 19–21].

**Fig. 1:**
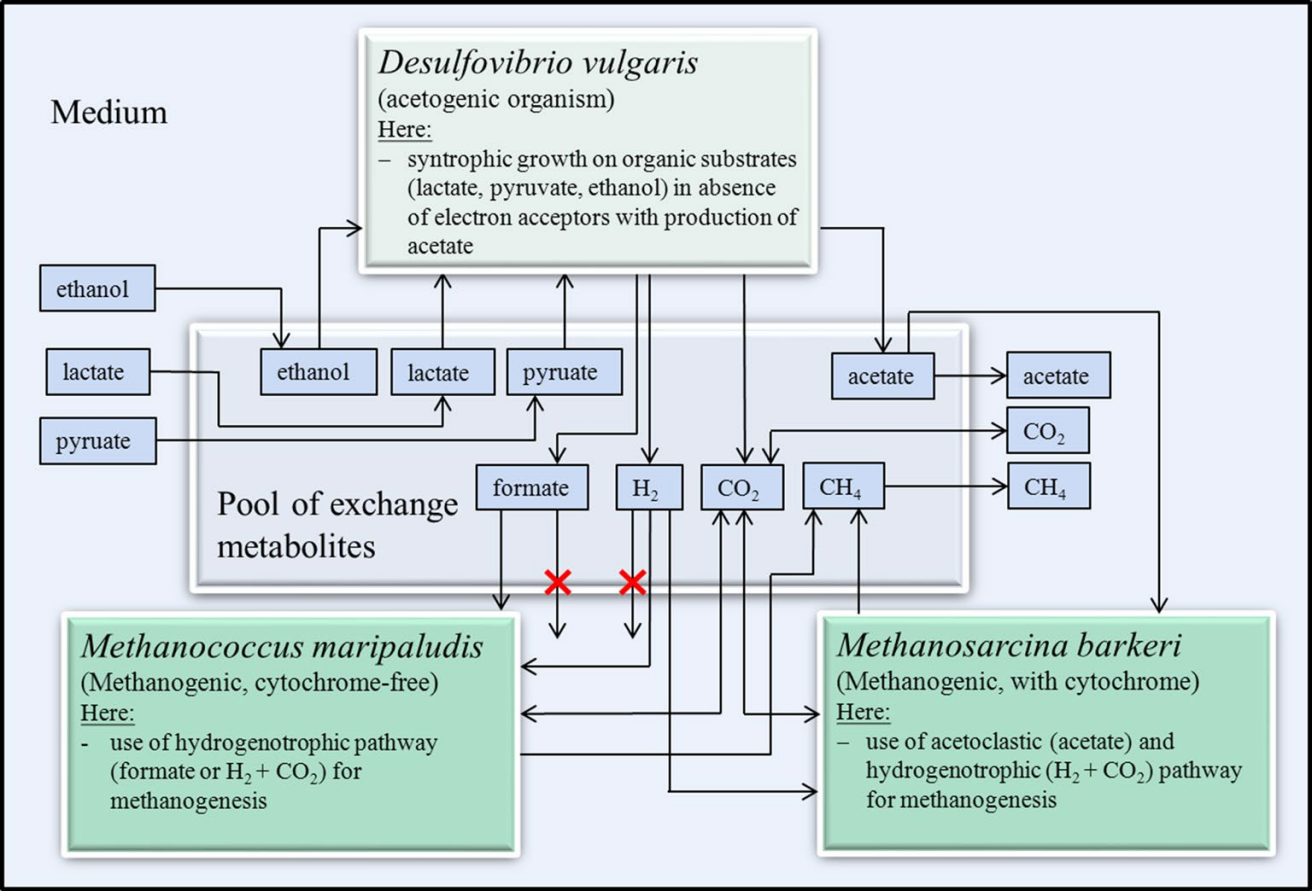
Outline of the community model for the last two steps of the biogas process (acetogenesis and methanogenesis) consisting of *D. vulgaris*, *M. maripaludis,* and *M. barkeri*. Production of hydrogen or formate by *D. vulgaris* is energetically favorable only for low hydrogen and formate concentrations and it is thus assumed that no net production of hydrogen and formate takes place in the community

In this study, we investigate a two-species community consisting of *D. vulgaris* and *M. maripaludis*, and a three-species community additionally taking into account *M. barkeri* (Fig. 1). The organisms chosen are involved in the last two stages of anaerobic digestion [22], and each organism represents one functional group in our model (*D. vulgaris*: acetogenic organism, *M. maripaludis*: hydrogenotrophic methanogenic without cytochrome; *M. barkeri*: acetoclastic and hydrogenotrophic methanogenic with cytochrome). Acetogenic microorganisms and methanogenic microorganisms live in a mutualistic (syntrophic) community [23]. The production of hydrogen or formate is only energetically favorable for low hydrogen and formate concentrations [23]. Thus, the acetogens need the methanogens to keep these concentrations low, while the methanogens on the other hand need the acetogens as they produce hydrogen, formate, and acetate utilized as substrates by the methanogens. While some dynamic models of anaerobic digestion include different groups of organisms (e.g., Anaerobic Digestion Model No 1 [24]), they usually neglect the metabolic flexibility of the involved species. In contrast, constraint-based modeling may account for all metabolic pathways of the community members. Stoichiometric two-species community models of anaerobic digestion were investigated by Stolyar et al. [11] as well as by Zomorrodi and Maranas [13]. Herein we will extend the scope of these models by including a third organism allowing also analysis of competitive interactions between methanogens.

The single-species models are the building blocks and therefore crucial for a functioning community model. Therefore, the first part of this study deals with the construction and validation of the single-species models with data from the literature. For assembling the community models, we use a compartmented approach and assume that all organisms grow with equal specific growth rates in a stable continuous process. This concept of balanced growth was first introduced by Khandelwal et al. [14] for stoichiometric community models and is a requirement for a stable community composition. We show how the modeling approach of Khandelwal et al. can be simplified such that standard simulation tools for constraint-based modeling can straightforwardly be used with the resulting models.

One central question in stoichiometric modeling of microbial communities is which objective function might be suitable to predict the metabolic behavior and composition of communities. Examples are maximization of the community growth rate or of the total biomass yield. Zomorrodi and Maranas [13] introduced a multi-level optimization approach with an inner objective (species level) and an outer objective (community level) functions. Herein we introduce a novel hierarchical optimization approach which involves two objectives (maximization of the community growth rate and of the biomass yield for each organism) and facilitates refined predictions on community metabolism and composition.

With a two-species and a three-species community model of the three representative organisms, we investigate syntrophic relationships as well as the influence of different substrates and of the ATP maintenance coefficients (ATPmaint) on the predicted community composition. We will also study which compositions are optimal in terms of methane yield and methane production rate. Finally, competition scenarios between the methanogenic organisms will be analyzed in the three-species community.

## Results and discussion

We constructed the single-species and community models as described in the “Methods” section. The size of the reconstructed networks is given in Table 1. Note that the number of metabolites and reactions in the community models is higher than the sum of reactions and the sum of metabolites of the single-species models because of the additional metabolite exchange pools and the reaction for the total biomass production (Eq. 5). In the following, we first describe and validate the single-species models before analyzing the community models.

**Table 1:** Model sizes of the single-species and community models

Model	Number of reactions	Number of metabolites
*D. vulgaris* (single-species)	114	99
*M. maripaludis* (single-species)	102	95
*M. barkeri* (single-species)	104	96
Two-species model (*D. vulgaris* + *M. maripaludis*)	220	202
Three-species model (*D. vulgaris* + *M. maripaludis* + *M. barkeri*)	328	301

A documentation and a separate SBML file for each model are given in the Additional files 2, 3, 4, 5, 6, and 7

### Single-species models

#### *M. maripaludis* and *M. barkeri*

*M. maripaludis* belongs to the group of cytochrome-free methanogens, while *M. barkeri* expresses cytochrome. The main differences between both groups are different ATP yields, a different affinity to hydrogen during hydrogenotrophic growth [25], and the use of different substrates for methanogenesis. Cytochrome-free methanogens have a higher affinity to hydrogen but a lower ATP yield. Thauer et al. [25] reviewed the differences in energy conservation in detail and we incorporated the pathways in the models accordingly. *M. barkeri* can use the acetotrophic, hydrogenotrophic, and methylotrophic pathway for methanogenesis, while *M. maripaludis* uses the hydrogenotrophic pathway only. Acetate has not been shown to support growth or methanogenesis in *M. maripaludis* [26]. *M. maripaludis* can also use formate as a substrate [26] which cannot be utilized by *M. barkeri* (Fig. 1). The two products released by both organisms are methane and CO_2_.

The models account for differences in the central metabolism of the two methanogens. Both use the acetyl-CoA pathway to produce acetyl-CoA from formate or CO_2_ and H_2_ [27]. *M. barkeri* possesses the oxidative and *M. maripaludis* the reductive branch of the tricarboxylic acid cycle (TCA) [27]. Furthermore, *M. maripaludis* uses the non-oxidative and *M. barkeri* the oxidative pentose phosphate pathway [27]. *M.barkeri* also uses a ribulose monophosphate pathway [27].

#### *D. vulgaris*

*D. vulgaris* is a sulfate-reducing organism that cannot oxidize acetate under anaerobic conditions. It can use lactate, ethanol, and pyruvate as sole carbon and energy source [28] as well as acetate as carbon source with hydrogen and sulfate as electron donor and acceptor [29]. *D. vulgaris* can also use alternative electron acceptors like thiosulfate, sulfite, nitrate and nitrite [29] (not considered herein). In the absence of electron acceptors, the organism can grow syntrophically with methanogens. Furthermore, *D. vulgaris* has an incomplete TCA and no non-oxidative pentose phosphate pathway [30].

Stolyar et al. [11] established a core model for *D. vulgaris* which was later extended by Zomorrodi and Maranas [13]. Both models do not support growth on ethanol without sulfate but different *Desulfovibrio* species can grow on ethanol without sulfate in syntrophic co-culture [31, 32]. We therefore included electron transport processes allowing growth of *D. vulgaris* under these conditions. There are several oxidation steps involved in utilization of ethanol, lactate, and pyruvate including various electron acceptors (e.g., ferredoxin and NADH). Oxidation of the electron acceptors may yield hydrogen or formate. Depending on the redox potentials of the reactions, the oxidation steps may be coupled with translocation of protons, either energetically uphill (protons are pumped outside) or downhill (protons flow back from the periplasm). We included three different electron acceptors in the model: Ferredoxin, NAD^+^, and a heterodisulfide (RS). Ferredoxin oxidation can be coupled with proton-pumping via EcH hydrogenase [Eq. (R1)] or RnF-complex [Eqs. (R1, R2)] [33–35], the oxidation of the heterodisulfide is coupled to the influx of protons (Eq. R4), and for NADH we considered a bifurcation mechanism that transfers electrons to ferredoxin and heterodisulfide (Eq. R3) [33, 36, 37]. During lactate oxidation, electrons are transferred to heterodisulfide. Furthermore, we assume that the electron acceptor NAD^+^ is used for the oxidation of ethanol to acetaldehyde, while electrons from acetaldehyde are transferred to a ferredoxin (acetaldehyde oxidoreductase):

R1 $$1 {\text{ Fd}}_{\text{red}} \leftrightarrow 1 {\text{ Fd}}_{\text{ox}} + {\text{ 1 H}}_{ 2} + \, ?{\text{ H}}^{ + } \left( {\text{ex}} \right)$$

R2 $$1 {\text{ Fd}}_{\text{red}} + {\text{ 1 CO}}_{ 2} \leftrightarrow 1 {\text{ Fd}}_{\text{ox}} + {\text{ 1 formate }} + \, ?{\text{ H}}^{ + } \left( {\text{ex}} \right)$$

R3 $$1 {\text{ NADH }} + \, 0. 5 {\text{ Fd}}_{\text{ox}} + \, 0. 5 {\text{ RS}} \leftrightarrow 1 {\text{ NAD}}^{ + } + \, 0. 5 {\text{ Fd}}_{\text{red}} + \, 0. 5 {\text{ RSH}}_{ 2}$$

R4 $$1 {\text{ RSH}}_{ \, 2} \,+ \, ?{\text{ H}}^{ + } \left( {\text{ex}} \right) \leftrightarrow 1 {\text{ RS }} + {\text{ H}}_{2}$$

The involved proton translocation processes and their stoichiometries are still not fully elucidated (question marks in Eqs. R1, R2, and R4). Therefore, we run simulations with different stoichiometries for translocated protons for the reactions R1, R2, and R4. We found that a stoichiometry of one translocated proton per ferredoxin oxidation and two translocated protons per heterodisulfide oxidation represented the experimental observation best. A detailed description with the simulation results and reasons for the decisions on the chosen stoichiometries are given in Additional file 1.

#### Model validation of single-species models

Table 2 summarizes the calculated maximum yields for ATP, biomass, and methane for the single-species models for growth on different substrates. The estimated maximum ATP and product yields reflect the expected values (according to biological knowledge). Calculated maximum biomass yields were close to experimentally determined biomass yields reported in the literature for the growth of *M. barkeri* on methanol and for *M. maripaludis* on formate. Validation for autotrophic growth on hydrogen plus CO_2_ was not possible. The maximum biomass yields are typically assumed for substrate-limiting conditions. In the reviewed literature, there were no substrate-limiting conditions (typically growth conditions were under gassing with 80 % H_2_ and 20 % CO_2_). Under these conditions yield suboptimal growth has been described [38]. For the growth of *D. vulgaris* on acetate with hydrogen and sulfate, the maximum biomass yield from the model agreed well with the experimental data from Badziong, Thauer [29] as well as from Nethe-Jaenchen, Thauer [39].

**Table 2:** Maximum (calculated from model) and experimentally determined yields for the growth of *M. maripaludis*, *M. barkeri*, and *D. vulgaris* on different substrates

Organism	Substrate	Max. methane yield (model) (mol/mol)	Max. ATP yield (model) (mol/mol)	Max. biomass yield (model) (gDW/mol)	Max. biomass yield per methane produced (model) (gDW/mol)	Max. biomass yield (literature data) (gDW/mol)	ATPmaint (literature data) [mmol/(gDW h)]	References
*M. barkeri*	Acetate	1	1.25	11.71	17.5	9.15	3.6	[50–52]
	Methanol	0.75	0.625	5.14	16.3	5.95	1.4	[50–53]
	CO_2_ + H_2_	1	1.5	9.73	8.2	–	–	
*M. maripaludis*	Formate	0.25	0.125	1.32	6.0	1.45	0.9	[54]
	CO_2_ + H_2_	1	0.5	4.78	6.0	–	–	
*D. vulgaris*	Acetate + H_2_ + sulfate	–	1	12.1		12.2	5.5	[29, 39]
						12.7	1.8; 5.6	

In addition, the ATPmaint coefficient calculated from the respective experiment is shown. The yields refer to the used substrate (in case of CO_2_ + H_2_, the yield refers to CO_2_). Note that the maximum biomass yields (model and experimental) refer to the (true) biomass yields, i.e., substrate consumption required for ATP maintenance is excluded when calculating the yield

We also used the experimental literature data to calculate the respective ATP maintenance coefficients (ATPmaint; Table 2; see also “Methods”). By averaging the ATPmaint coefficients for the different substrates and for each organism, we obtained the following values which will later be used in the community simulations:

- *D. vulgaris*: 4.3 mmol/(gDW h);
- *M. maripaludis*: 0.9 mmol/(gDW h);
- *M. barkeri:* 2.5 mmol/(gDW h).

Furthermore, from the maximum growth rates observed in the experiments, we estimated the maximum product formation rates which were also integrated as upper bounds in the single-species (and community) models:

- *D. vulgaris*: 50 mmol acetate/(gDW h);
- *M. maripaludis*: 15 mmol methane/(gDW h); and
- *M. barkeri*: 15 mmol methane/(gDW h).

### Simulation results of the two-species community model

**Fig. 2:**
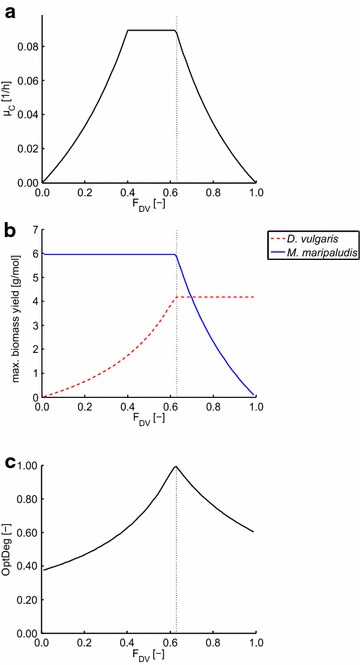
Maximum community growth rate, biomass yields, and optimality degree in the two-species model for growth on lactate. **a** Maximum community growth rate *µ*_*C*_ as function of the community composition (*F*_DV_: biomass fraction of *D. vulgaris*; the fraction of *M. maripaludis* is 1-*F*_DV_) in the two-species model with lactate as substrate for *D. vulgaris*. **b** Maximum biomass yields for *D. vulgaris* (per lactate consumed; *red dashed line*) and for *M. maripaludis* (per methane produced, *blue solid line*). **c** Optimality degree (OptDeg) of the community versus fractional biomass abundance. The maximum OptDeg gives the predicted operation point. The biomass yields (**b**) and OptDeg (**c**) were calculated for the maximum community growth rate at the respective community composition

In a first FBA simulation (see “Methods”), we calculated the maximum community growth rate of the two-species community composed of one acetogen (*D. vulgaris*) and one methanogen (*M. maripaludis*) for different community compositions for growth on lactate. *D. vulgaris* consumes lactate and produces acetate as well as carbon dioxide and hydrogen or/and formate in this community. *M. maripaludis* consumes the produced hydrogen, carbon dioxide, and formate, while producing methane. The acetate produced by *D. vulgaris* is not consumed by *M. maripaludis* and thus accumulates in the medium. Initially, for illustration purposes, we set the ATPmaint coefficient to zero for both species. The results are shown in Fig. 2a for growth on lactate. There is a broad range from 40 to 63 % of relative biomass abundance of *D. vulgaris* in which the community reaches its maximum growth rate of 0.089 h^−1^. At this point, growth becomes limited by the maximum methane production rate of *M. maripaludis*.

In order to understand how the two species must behave under the different biomass compositions to facilitate balanced growth of the community, we analyzed the maximum biomass yields for both organisms that are possible under maximum growth for the respective biomass compositions (Fig. 2b). The biomass yield of *D. vulgaris* refers to the substrate lactate. In contrast, for the biomass yield of *M. maripaludis*, we relate the biomass synthesized to the methane produced as *M. maripaludis* can use two substrates (hydrogen plus CO_2_ or formate). Relating the biomass yield, for instance, to the hydrogen consumption rate could result in infinite biomass yields if formate instead of hydrogen is used as substrate. However, both substrates can be converted to methane and the biomass yield per methane produced is equal for both substrates (see Table 2).

As long as the biomass fraction of *D. vulgaris* (*F*_DV_) is lower than 0.63, this organism grows with suboptimal biomass yield because it converts larger amounts of lactate to fermentation products (acetate as well as hydrogen and carbon dioxide or formate) instead of own biomass to support the substrate requirements of *M. maripaludis* present in high abundance (*F*_MM_ > 0.37). This even holds for the case where the community grows with maximum community growth rate (*µ*_*C*_ = 0.089 h^−1^; 0.40 < *F*_DV_ < 0.63). Hence, for *F*_DV_ < 0.63, *D. vulgaris* would behave “altruistically” and grow with suboptimal biomass yield to keep the community in a balanced state (see red line in Fig. 2b). In contrast, *M. maripaludis* can grow with maximal biomass yield under these conditions and thus behave “selfish” (blue line in Fig. 2b). There is only one point at *F*_DV_ = 0.63 (*F*_MM_ = 0.37), where both organisms reach their respective maximum biomass yields. For *F*_DV_ > 0.63, the opposite behavior can be seen: now, the smaller population of *M. maripaludis* must behave “altruistically” and grow with suboptimal biomass yield to allow the large population of *D. vulgaris* to grow. More precisely, *M. maripaludis* would need to consume large amounts of hydrogen or formate produced by *D. vulgaris* and waste the thereby generated ATP in order to balance the whole community at the respective composition.

Even though a plateau of maximum community growth rates for different biomass fractions exists, we argue that the single point (*F*_DV_ = 0.63, *F*_MM_ = 0.37) where both organisms grow with maximum biomass yield and can thus behave “selfish” will be the final attractor of this system. As long as *F*_DV_ < 0.63, the community will not stay in steady state as *D. vulgaris* will instead increase its growth rate by a more efficient use of the substrate resulting in a higher relative biomass abundance of this species. Likewise, for *F*_DV_ > 0.63 (*F*_MM_ < 0.37), we expect that *M. maripaludis* would increase its growth rate and therefore its biomass fraction in the community. We therefore propose a hierarchical optimization approach to predict community compositions by using “maximization of community growth rate” as primary and “maximization of biomass yield” as secondary objective (see “Methods”). To quantify the optimality of a community with respect to the overall biomass yield (secondary objective), we introduce the optimality degree (OptDeg) as described in the “Methods” section [Eqs. (7) and (8)]. OptDeg integrates terms of the biomass yields of each species population and relates it to the theoretically feasible maximum. Therefore, in the considered two-species model, OptDeg reaches its maximum one if both populations operate with maximum biomass yields, hence where *F*_DV_ = 0.63 (Fig. 2c).

Following the argumentation above, we thus predict that the biomass composition where all species grow with maximum specific growth rate *and* with maximum biomass yields (i.e., where OptDeg = 1) will be the stable point of operation of the community. Hence, in the two-species example, this criterion reduces the possible range of optimal community compositions to a single point. As we will see later on, this may change in the case of competition in a three-species model.

**Fig. 3:**
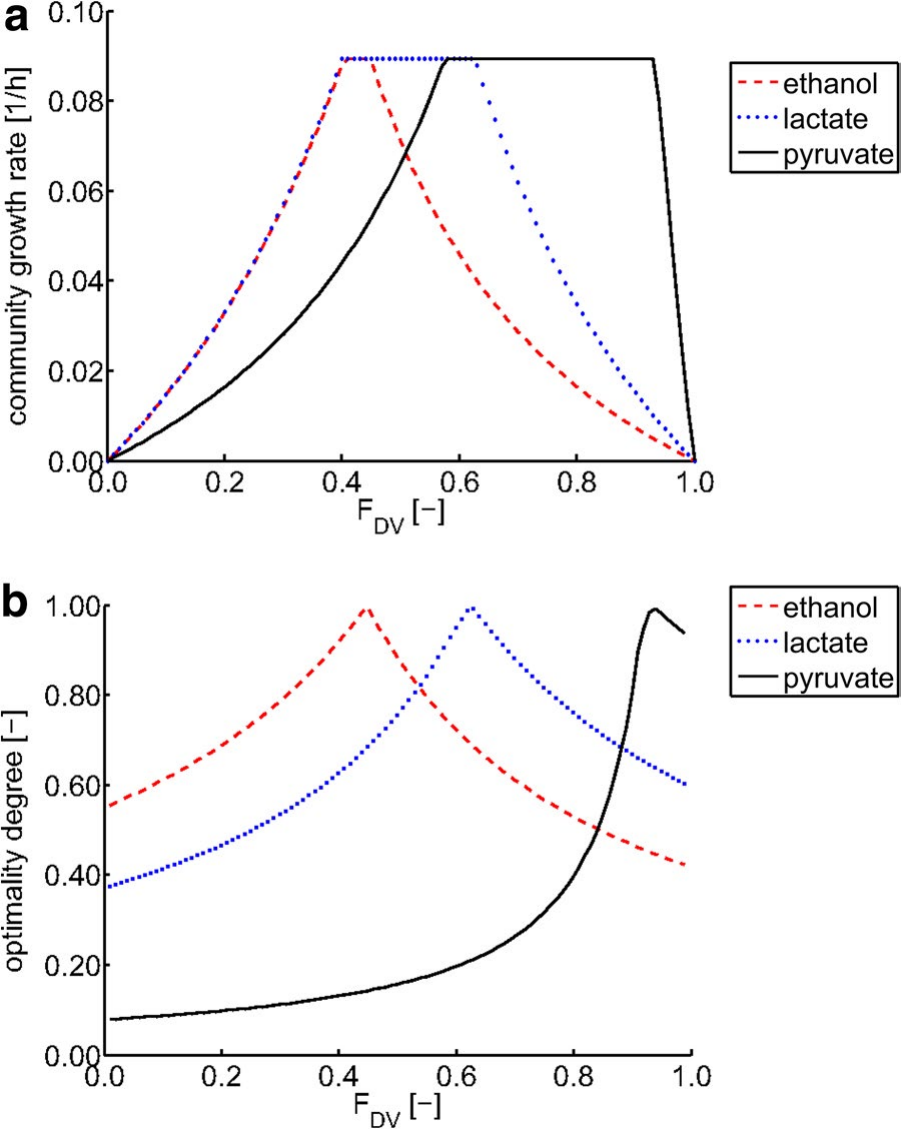
Maximum community growth rate and optimality degree in the two-species model for growth on ethanol, lactate, and pyruvate. **a** Maximum community growth rate as function of the community composition (*F*_DV_: biomass fraction of *D. vulgaris*; the fraction of *M. maripaludis* is 1-*F*_*DV*_) in the two-species model for growth on ethanol (*red*, *dashed*), lactate (*blue*, *dotted*), and pyruvate (*black*, *solid*) versus relative biomass abundance of *D. vulgaris*. **b** Optimality degree (OptDeg) for the two-species model for growth on ethanol (*red*, *dashed*), lactate (*blue*, *dotted*), and pyruvate (*black*, *solid*) versus relative biomass abundance of *D. vulgaris*. The maximum OptDeg gives the predicted operation point. The OptDegs were calculated for the respective maximum community growth rates for the different biomass compositions (see also Fig. 2)

Similar simulations were also performed for growth on pyruvate and ethanol. The results are shown in Fig. 3 indicating that the maximum community growth rate is the same for all substrates, whereas the predicted community composition (OptDeg = 1) is governed by the substrate. For growth on pyruvate, the predicted relative abundance of *D. vulgaris* is 92 %, whereas for growth on ethanol it reduces to 42 %. This can be explained as follows: in the model, *D. vulgaris* has different maximum biomass yields [g_DW_/mmol substrate] for the various substrates (Y_X/pyruvate_ > Y_X/lactate_ > Y_X/ethanol_, see Additional file 1: Table S1). Additionally, the yield of cross-feeding metabolites (hydrogen and formate) is twice as high for ethanol and lactate compared to pyruvate, which explains the shifts in the predicted community composition. Also, the width of the range of community compositions with maximum community growth rate differs for the substrates (smallest for ethanol and broadest for pyruvate). For substrates with high biomass yields (e.g., pyruvate), the organism has more room to waste energy while still being able to reach the maximum growth rate which is limited by *M. maripaludis* (see above).

To investigate the influence of the ATPmaint coefficients on the community composition, we next simulated scenarios with different maintenance coefficients (growth on lactate):

- 0 mmol_ATP_/g_DW_/h (neglecting the demand of ATP for maintenance metabolism as in the simulations shown in Figs. 2 and 3).
- Equal maintenance coefficient for both organisms (3 mmol_ATP_/g_DW_/h).
- Different maintenance coefficients as derived from the literature data (see above; ATPmaint = 0.9 mmol_ATP_/g_DW_/h for *M. maripaludis* and ATPmaint = 4.3 mmol_ATP_/g_DW_/h for *D. vulgaris*).

**Fig. 4:**
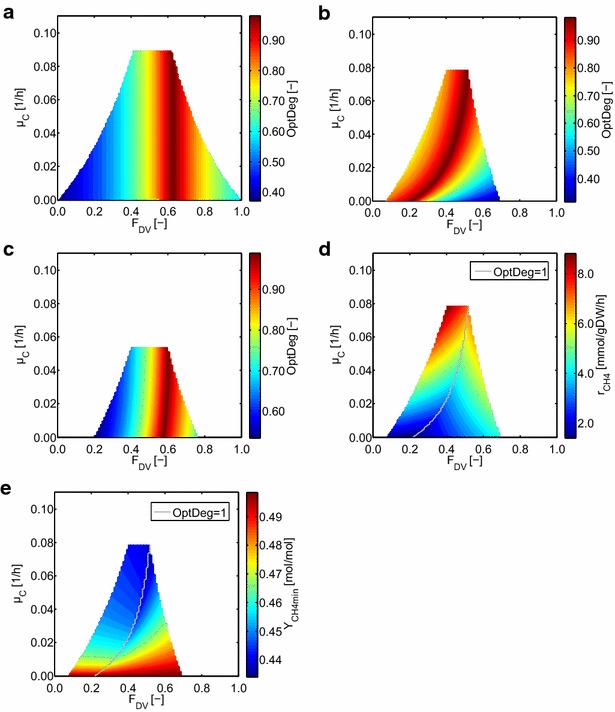
Optimality degree OptDeg for different growth rates and community compositions of the two-species model (with lactate as substrate for *D. vulgaris*) for different maintenance coefficients. **a** 0 mmol_ATP_/gDW/h for both organisms, **b** 4.3 mmol_ATP_/gDW/h for *D. vulgaris* and 0.9 mmol_ATP_/gDW/h for *M. maripaludis*, and **c** 3 mmol_ATP_/gDW/h for both organisms. For case **b**, the minimum methane production rates (**d**) and the minimum methane yields referred to lactate (**e**) are shown

In these simulations, we calculated OptDeg for all possible (not only the maximum) community growth rates for a given biomass composition to consider also cases where the maximum community growth rate cannot be reached, for example, due to substrate or nutrient limitations in a chemostat. The results are shown in Fig. 4.

We first observed that the optimal community composition is independent of the growth rate in simulations that do not take the maintenance coefficient into account. In contrast, simulations with non-zero ATPmaint coefficients show not only smaller maximum community growth rates but also a pronounced dependency of the community composition with respect to the growth rate. Changes in the relative species abundances depend on the ratio of the maintenance coefficients of the organisms. For equal ATPmaint coefficients, optimal community compositions with maximal biomass yields (OptDeg = 1) can again be seen at approximately *F*_DV_ = 0.63 for all growth rates, while different maintenance coefficients (reflecting the values estimated from the literature data) result in a significant shift of the community composition. With the maintenance coefficients estimated from the literature data, the predicted relative biomass abundance for *D. vulgaris* is only 30 % for smaller growth rates and increases up to 50 % at the maximum growth rate (approx. 0.09 h^−1^). Generally, the maintenance coefficients affect the specific biomass yields (increases with increasing growth rate) and thus the community composition, especially under low growth rates. Therefore, the maintenance coefficient plays a pivotal role for microbial communities especially for the typically low growth rates that are found in anaerobic processes. ATPmaint is not a constant parameter but depends on environmental factors including weak acid stress, temperature [40], nitrogen source [41], electron acceptors, and substrates [38, 39, 42, 43]. Accordingly, ATPmaint needs to be carefully determined to quantitatively describe metabolic dependencies between the species of the community.

For the scenario, where we used the estimated values for ATPmaint coefficients (Fig. 4b), we also calculated the minimum methane production rates (Fig. 4d) and the minimum methane yields referred to lactate (Fig. 4e). In general, the specific methane production rate increases with the growth rate because more substrate needs to be converted to produce higher amounts of biomass. For a fixed growth rate, we see that the specific methane production rate is the lowest where OptDeg is maximal and increases with decreasing OptDeg (Fig. 4d). This can be explained by the fact that, for lower values of OptDeg, one of the organisms grows with suboptimal biomass yield and thus consumes more substrate per biomass produced. In the model, a larger fraction of the substrate taken up is used to produce extra amounts of ATP (which is then wasted in futile cycles of the metabolism of the respective species). Consequently, this results in the production of more methane (if *M. maripaludis* grows with suboptimal biomass yield) or more byproducts (*D. vulgaris*) which in turn must be metabolized by *M. maripaludis* and, again, increases the methane production rate per consumed lactate. Similarly, minimal methane yields are (for constant growth rates) lower in areas with a high OptDeg and higher where the OptDeg is low. However, in contrast to the methane production rate, the simulations show high-methane yields for low growth rates because the fraction of substrate used for producing ATP for maintenance processes is then higher compared to high growth rates.

#### Comparison with experimental data from the literature

Meyer et al. cultivated *D. alaskensis*, which is represented by the closely related organism *D. vulgaris* in our model, in co-culture with *M. maripaludis* or *M. hungatei* in continuous culture on pyruvate and lactate. With pyruvate as a substrate *D. alaskensis* dominated the co-culture, while the community consisted of almost equal amounts of both organisms in lactate medium [36]. The estimated specific flux rates and mass ratios of the organisms for two different growth rates as well as the simulation results for community composition and the corresponding substrate uptake and product formation fluxes are given in Table 3. Concentrations of carbon monoxide and succinate were low and thus neglected for the simulations. The predictions made by the model reflect the experimental data very well for the lower growth rate of 0.027 h^−1^. However, the simulated absolute fluxes are generally lower for the higher growth rates, while the ratios of the fluxes are similar. This indicates that the theoretical biomass yields predicted by the model are too optimistic, at least for higher growth rates. Changing biomass compositions at higher growth rates might be one possible reason. On the other hand, due to changes of product concentrations in the medium that may, for example, lead to weak acid stress, a higher growth rate could also indirectly lead to an increase in maintenance coefficients (and thus to a reduced biomass yield). Regarding the community composition, good agreements with the model predictions can be observed for at least three of the four cases. The shift in the community composition from almost equal amounts of both organisms in lactate medium to a *D. vulgaris*-dominated culture in pyruvate medium is reflected by the simulations and can, as described above, be explained by the fact that the biomass yield of *D. vulgaris* is higher, while the yield of hydrogen and formate is lower for pyruvate.

**Table 3:** Estimated flux rates and steady-state biomass compositions from Meyer et al. [36, 48] for growth of *D. alaskensis* with *M. maripaludis* or *M. hungatei* on pyruvate and lactate medium [36]

Substrate	Growth rate (h^−1^)	Specific flux rates (mmol/g_DW_/h)			*F*_DV_ (−)
		r_pyruvate/lactate_	r_acetate_	r_methane_	
Pyruvate	0.027	6.6–7.4	5.9–6.9	1.6–1.8	0.79
		(*5.36*)	(*5.02*)	(*1.09*)	(*0.83*)
	0.047	11.4–12.9	10.5–11.9	2.7–3.0	0.77
		(*6.99*)	(*6.36*)	(*1.36*)	(*0.86*)
Lactate	0.027	9.13	8.22	4.34–5.79	0.39
		(*8.17*)	(*8.00*)	(*3.67*)	(*0.42*)
	0.047	16.47	14.82	7.49–8.99	0.47
		(*11.60*)	(*11.26*)	(*5.12*)	(*0.48*)

Flux rates for hydrogen, carbon monoxide, and succinate were not considered for the simulations because they had only very small values (<0.2 mmol/g/h for succinate and <0.005 mmol/g/h for H_2_ and CO). The model predictions are given in italics in brackets. We used ATPmaint = 4.3 mmol_ATP_/gDW/h for *D. vulgaris* and ATPmaint = 0.9 mmol_ATP_/gDW/h for *M. maripaludis.* The predicted community composition (*F*_DV_) was determined by fixing the growth rate to the value of the experiment and then taking the composition with OptDeg = 1

In the experiments performed by Tatton et al. [31], methane yields were estimated for a two-species culture related to the one considered herein. For a dilution rate (growth rate) of 0.042 h^−1^, they measured a methane yield of 0.45 mol methane/mol ethanol. To compare these results with model predictions, we fixed *µ*_*C*_ in the simulations to 0.042 h^−1^ and calculated the methane yield at the optimal operation point (OptDeg = 1). The resulting predicted methane yield was 0.448 mol/mol which thus agrees almost perfectly with the yield reported by Tatton et al. [31].

### Simulation results of the three-species community model

For the three-species model, we added the *M. barkeri* compartment resulting in a community similar to the one studied in Tatton et al. [31] growing on ethanol in sulfate-free medium. We investigated three different dependency scenarios for growth on ethanol: (1) competition for hydrogen, (2) usage of alternative substrates (hydrogen and acetate), and (3) a combination of both. We used the ATPmaint coefficients as estimated from the literature data (see above).

**Fig. 5:**
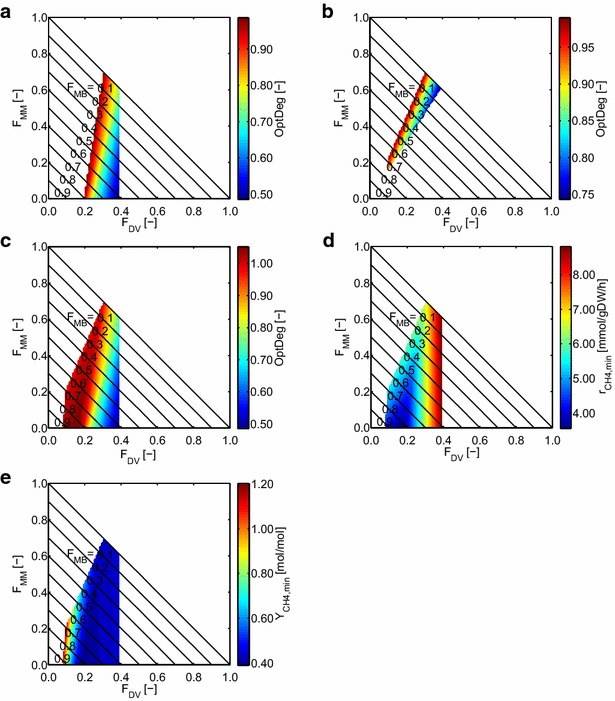
Simulations with the three-species model (*D. vulgaris, M. maripaludis, and M. barkeri*) for growth on ethanol. **a**–**c** show optimality degree OptDeg for the maximum community growth rate (0.0505 h^−1^) calculated for different community compositions. Three scenarios were simulated: **a** competition scenario: both methanogens can only use the hydrogenotrophic pathway for methanogenesis. **b** Use of hydrogenotrophic pathway for *M. maripaludis* and acetoclastic pathway for *M. barkeri* **c** use of hydrogenotrophic pathway for *M. maripaludis* and hydrogenotrophic and acetoclastic pathway for *M. barkeri. F*_DV_: biomass fraction of *D. vulgaris*; *F*_MM_: biomass fraction of *M. maripaludis*. We used ATPmaint = 4.3 mmol_ATP_/gDW/h for *D. vulgaris*, ATPmaint = 0.9 mmol_ATP_/gDW/h for *M. maripaludis* and ATPmaint = 2.5 mmol_ATP_/gDW/h for *M. barkeri*. For scenario **c** the minimum methane production rates (**d**) and minimum methane yield (referred to ethanol) (**e**) were also calculated

Figure 5a shows the simulation result of the competition scenario for the maximum community growth rate. A straight line between 18 % *D. vulgaris*/82 % *M. barkeri*/0 % *M. maripaludis* and 30 % *D. vulgaris*/*0* *% M. barkeri*/70 % *M. maripaludis* represents the predicted community composition (OptDeg = 1). None of the two methanogens is preferred. This result is to be expected because both methanogens have the same function in this scenario (hydrogen utilization) and should thus be equivalent. Nevertheless, in real settings, it can be assumed that factors like the hydrogen concentration, the pH value, or the temperature can lead to the dominance of one of the methanogens. With an increasing percentage of *D. vulgaris*, the OptDeg decreases. For *F*_DV_ > 40 %, the maximum community growth rate is not reached anymore. The fact that the line with OptDeg = 1 is on the left side of the solution space (low amounts of *D. vulgaris*) indicates that the community is limited by *D. vulgaris* in this scenario. Obviously, *D. vulgaris* is essential in the community and can live with either or both methanogens.

In the second scenario, *M. barkeri* uses acetate instead of hydrogen as the only substrate (Fig. 5b). There is a band of OptDeg = 1 between *F*_DV_ = 30 %/*F*_MM_ = 70 %/*F*_MB_ = 0 % and *F*_DV_ = 10 %/*F*_MM_ = 20 %/*F*_MB_ = 70 %. The OptDeg again decreases with increasing amounts of *D. vulgaris* and no solutions (with maximum community growth rate) exist left to the band of OptDeg = 1 (lower amounts of *D. vulgaris*). Also, no solutions exist for a fraction of *M. maripaludis* below 20 % or *D. vulgaris* above 40 %. *M. maripaludis* becomes essential for the community in this scenario because it is the only hydrogen consumer. *M. barkeri* on the other hand is optional because accumulation of acetate in the medium is possible and acetate degradation is therefore not required. Compared to the competition scenario (Fig. 5a), the methanogens can reach a higher relative biomass abundance [overall 90 % (Fig. 5b) compared to 80 % in the competition scenario Fig. 5a)] which is to be expected because acetate can be used as an additional substrate for methanogenesis.

In the last scenario, *M. barkeri* can use both, acetate and hydrogen, as substrates (Fig. 5c). Interestingly, there is a small area with an OptDeg greater than unity (up to 1.066). In this special case (which can only arise in scenarios with more than one substrate per species), the biomass yield of the substrate combination (acetate and hydrogen) is higher than the sum of the maximum biomass yields for the single substrates for *M. barkeri.* Here we can still assume that solutions with OptDeg ≥ 1 are the optimal and preferred solutions because the organisms convert the substrate(s) taken up to biomass with maximum yield (no wasting of energy) in this case. Note also that the solutions for the predicted community composition include the solutions from the other two scenarios and all combinations of both. This results in a broader region of potential solutions.

For this scenario (Fig. 5c) we also calculated the minimum specific methane production rates (Fig. 5d) and the minimum methane yield for the use of ethanol (Fig. 5e). Again, areas with a low OptDeg have higher minimal methane production rates than areas with a high OptDeg. As stated for the two-species model, this can be explained by the wasting of energy in the areas with low OptDeg leading to higher methane synthesis rates. The minimum methane yields are low (0.4–0.5 mol/mol) for a fractional biomass abundance of 20–40 % of *D. vulgaris*. For decreasing amounts of *D. vulgaris*, the minimum methane yield strongly increases up to 1.2 mol/mol. Interestingly, in this area of maximum methane yield, the OptDeg is one (see Fig. 5c) indicating an optimal community composition with high-methane yield. The strong increase of the methane yield per ethanol for low percentage of *D. vulgaris* can be explained by the additional use of acetate for methanogenesis. In the area with *F*_DV_ > 0.2, the fraction of the methanogens is not high enough to use all available substrates (acetate, hydrogen, and formate) and since the accumulation of hydrogen and formate is not allowed, only the hydrogenotrophic pathway is used. In the region of optimal solutions (OptDeg ≥1), the right border (*F*_DV_ = 0.2) shows solutions were only the hydrogenotrophic way is used and thus the methane yield is below 0.5 mol/mol (which is the theoretical maximum methane yield if no acetate is consumed). At *F*_DV_ = 0.1, both hydrogen and acetate are completely used which results in high-methane yields per substrate (maximum theoretical methane yield is 1.5 mol/mol if all acetate is used for methane production).

#### Comparison with experimental data from the literature

In addition to the two-species culture (see above), Tatton et al. [31] also measured the methane yield for growth on ethanol in a three-species culture comparable to the one considered here. The culture consisted of a *Desulfovibrio* strain FR17 (acetogen, represented by *D. vulgaris* in the model), *Methanobacterium* strain FR2 (cytochrome-free methanogen, represented here by *M. maripaludis*), and *Methanosarcina mazei* (represented here by *M. barkeri*), where the latter was adapted to use acetate as the sole carbon and energy source [31]. The concentrations of acetate were low in the three-species culture (below 5 mM at steady state compared to 50 mM when the three-species culture was first initiated) [31]. We therefore chose the solutions from our simulations where all acetate was consumed by *M. barkeri*. Tatton et al. were able to obtain steady states up to a dilution rate of 0.033 h^−1^. In the range of dilution rates of their experiments (between 0.012 and 0.033 h^−1^), the measured methane yield was 1.42 mol/mol [31]. In our simulations, the predicted methane yield for this range of dilution rates lies between 1.23 and 1.37 mol/mol and is thus only slightly lower than the experimentally determined values. Again, this could be related to higher ATP maintenance costs which would increase the methane yield.

Unfortunately, Tatton et al. did not provide information on the community composition. And, in general, there seems to be only few experimental data for defined mixed cultures. Accordingly, our model and simulation approach might help to gain insights in metabolic constraints of those cultures also when respective experimental data are not available.

## Conclusions

Microbial communities play a major role in anaerobic digestion and our study demonstrates that the use of stoichiometric models can be a valuable tool to gain insights about factors influencing the composition and stability of these communities.

We first established single-species models of three different organisms involved in the last steps of anaerobic digestion. The single-species models are crucial for a functioning community model and thus we carefully constructed and validated those models with experimental data from the literature. In some cases (e.g., autotrophic growth of methanogens), validation was not possible due to a lack of suitable data and in those cases further experimental investigations are necessary. Furthermore, we suggest a mechanism for growth of *D. vulgaris* on ethanol in the absence of sulfate which needs experimental validation.

In a next step, we connected the single-species models in a compartmented approach to create a community model and applied for the first time the approach of balanced growth proposed by Khandelwal et al. [14] in the context of biogas production. While we adopted this framework, we simplified the model representation (see [Eqs. (4) and (5)] in the Methods section) allowing direct implementation and analysis of the resulting models by standard tools for stoichiometric modeling and simulation.

In order to predict community compositions, we proposed a hierarchical optimization approach based on two objectives: maximization of the community growth rate and, as secondary objective, optimal substrate usage (biomass yield) for each organism involved. Compared to maximization of the community growth rate alone, solutions with unrealistic “altruistic” behaviors of organisms in the community are excluded resulting in a much smaller range of predicted optimal community compositions; in case of the two-species model it becomes a single point, whereas lines or regions of optimality can arise if multiple organisms with similar (or even identical) metabolic functions exist in the community. This approach, as well as the whole methodology applied here, can readily be applied to more complex communities. Compared to OptCom [13], which relies on a multi-level optimization framework, our approach appears simpler because OptDeg, quantifying the degree of optimality with respect to biomass yield, can be easily calculated for any given growth rate by a linear optimization with a single objective function. A comparison of predicted community compositions with measurements from two-species experiments showed good agreement confirming the validity of our approach.

Our work extended the scope of the theoretical studies by Stolyar et al. [11] and Zomorrodi and Maranas [13] by constructing and analyzing for the first time a three-species model for anaerobic digestion including a second methanogenic organism (*M. barkeri*). This three-species model resulted in different options for the community (competition, use of different substrates). We showed that competition (two organisms using the same substrate, thus having the same function in the community) leads to exchangeability of the organisms. The three scenarios resulted in different predicted patterns of community compositions for which experimental data are not yet available for validation.

As one important result of our simulations, we showed that a different ATPmaint of the species have a major impact on the community composition, especially for low growth rates. Hence, maintenance coefficients, which may depend on environmental conditions, need to be carefully considered when studying slow metabolic processes like anaerobic digestion.

Importantly, our model also enabled us to give predictions at which community composition maximum specific methane production rates and yields can be expected. Apparently, maximum values for these key process parameters of biogas plants can only be reached if some species in the process waste substrate and energy. However, this would be accompanied by lower biomass yields, which is contrary to the objective of the involved species and would also negatively affect the volumetric productivity.

In the future, the next step will be to investigate more complex community models of anaerobic digestion by including more species. These models will help to gain a deeper understanding of the key characteristics of the biogas process and, as ultimate goal, might eventually be used to develop intervention or control strategies to improve the process in terms of product yields and stability. While the considerations made herein focused on the biogas processes with emphasis on product (methane) synthesis, our models may also shed light on metabolic interactions of these communities in their natural habitats.

## Methods

### Stoichiometric and constraint-based modeling

Stoichiometric and constraint-based metabolic modeling has become a standard tool to analyze key properties and capabilities of the metabolism of diverse organisms [8–10]. As a steady-state approach, it relies solely on the structure of metabolic networks and does not need kinetic parameters. Hence, this modeling approach is well suited especially for modeling large-scale systems including microbial communities. In stoichiometric network analysis, a steady state of metabolite concentrations is assumed where no net production or consumption of internal metabolites occurs. This is based on the observation that intracellular enzyme reactions are fast compared to changes in gene expression and dynamic changes in the environment [8]. The steady-state assumption leads to the metabolite balancing equation:

1 $$\bf {Nr}~ = ~{\bf{0}}$$

where *N* is the stoichiometric matrix that contains the stoichiometric coefficients of all reactions and *r* is the vector of net reaction rates. In addition, flux capacity constraints (lower and upper boundaries) for the reaction rates can be considered:

2 $$\it {lb}_{i} \le r_{i} \le {\it{ub}}_{i}.$$

In particular, for irreversible reactions, *r*_*i*_ ≥ 0 must be fulfilled. An important method frequently applied in stoichiometric modeling is FBA. FBA maximizes a linear objective function.

3 $$\mathop {\text{maximize}}\limits_{\bf{r}} \;\;\;\;\bf{c}^{T} \bf{r}$$

Subject to Eqs. (1) and (2), typical objective functions are the maximization of the biomass yield, growth rate, or product yield. Here we use FBA to calculate (i) the maximum community growth rate, (ii) the maximum biomass yields, and (iii) the OptDeg (see below).

### Single-species models

For construction of the single stoichiometric metabolic models of *D. vulgaris*, *M. maripaludis*, and *M. barkeri*, we used the KEGG [27] and MetaCyc [44] databases as well as information from a number of publications. Similar to Stolyar et al. [11], we constructed the single-species models with a focus on the central metabolism. The models contain the major pathways of energy, redox, and precursor metabolism, since these pathways govern the metabolic interdependencies between the organisms of the communities.

Stoichiometric metabolic models usually include a biomass synthesis reaction (BSR) specifying the stoichiometries of precursors, energy (ATP), and reduction equivalents (NADPH) needed to generate biomass of the organism investigated. Since detailed information about the specific biomass composition for the three species was not available and because moderate variations in the biomass compositions usually have a minor impact on major fluxes in the central metabolism, we used for all three organisms the BSR from the *Escherichia coli* model of Stelling et al. [45].

#### Maintenance coefficients

Using the experimental literature data and applying the method of Pirt [46], we calculated the (true) biomass yields and the substrate consumption rates for maintenance processes. From the latter, we calculated a lower bound for the non-growth-associated ATP maintenance (ATPmaint) coefficient by multiplying the substrate uptake rate at zero growth with the maximum ATP yield (calculated by the model) for this substrate in the respective organism.

### Community models

The model of each of the three species considered was included as a compartment in the community model (Fig. 1). An additional compartment for exchange of metabolites connects the species compartments: organisms were allowed to consume metabolites from and to excrete products into the pool of exchange metabolites via transport reactions. The pool of exchange metabolites was also connected to the medium to allow uptake of substrates and accumulation of some products in the environment.

For simulating the community, we decided to use the concept of balanced growth [14] demanding that all organisms must grow with the same specific growth rate to get a stable community composition. Balanced growth applies to microbial communities that function in a fairly constant environment and here we consider the biogas process (e.g., in a (quasi-) continuously operating biogas plant) as such. The assumption of balanced growth allows us to use constraint-based methods, where steady state is a key requirement [see Eq. (1)].

While we adopted the basic concept of Khandelwal et al. [14], we use a slightly different representation of the resulting stoichiometric community models simplifying their implementation and analysis in existing software tools for stoichiometric modeling. In a community model with *n* species, the model contains *n* species biomass compounds (BM_1_,…, BM_*n*_) as well as a compound representing the (total) community biomass (BM_*C*_). Each biomass compound BM_*i*_ is produced by the precursors p_*i*,1_,…,p_*i,k*_ (with stoichiometric factors α_*i,*1_,…, α_*i,k*_) to be generated by species *i*:

4 $$\alpha_{i,1} p_{i,1} + \alpha_{i,2} p_{i,2} + \cdots + \alpha_{i,k} p_{i,k} \to \left[ { 1 {\text{ gram}}} \right]{\text{ BM}}_{i}.$$

The total biomass compound BM_*C*_ is composed of the *n* species biomass compounds, each having a specific fraction *F*_*i*_ (gDW_*i*_/gDW_*C*_) at the total biomass.  **F ** = (*F*_1_,*F*_2_,…,*F*_*n*_) thus describes the community composition. Obviously, the fractions must fulfill $F_{1} + F_{2} + \cdots + F_{n} = 1$ . Consequently, formation of total community biomass can be described by

5 $$F_{1} \cdot {\text{BM}}_{ 1} + F_{2} \cdot {\text{BM}}_{ 2} + \cdots + F_{n} \cdot {\text{BM}}_{\text{n}} \to \left[ { 1 {\text{ gram}}} \right]{\text{ BM}}_{C}.$$

BM_*C*_ is considered as external metabolite and may thus accumulate in the model. The rate of Eq. (5) is the community growth rate *µ*_*C*_ (h^−1^), whereas the rates of the reactions in Eq. (4) are the specific biomass production rates *r*_BM*i*_ given in (gDW_*i*_/gDW_*C*_/h). Note that in steady state it must hold that *r*_BM*i*_ = *F*_*1*_*∙µ*_*C*_. Hence, although the effective growth rate of all species is identical (*µ*_*C*_), different biomass fractions can exist implying different amounts of biomass produced by the species. All fluxes in the model refer to total biomass and all fluxes except *µ*_*C*_ and *r*_BM*i*_ have unit (mmol/gDW_*C*_/h). Therefore, specific constraints (e.g., maximum substrate consumption or maintenance coefficient of organism *i*) must be multiplied with *F*_*i*_ to correctly reflect capacities.

With this procedure, assembly of a stoichiometric community model from the single-species models is straightforward. One basically needs to (i) introduce the pools of exchange metabolites, (ii) define the biomass composition *F*, (iii) introduce reaction Eq. (5) for the total biomass, and (iv) relate specific fluxes (or flux bounds) to the total biomass. The names of internal metabolites must differ in the species models, e.g., pyruvate_1_, pyruvate_2_, …, pyruvate_*n*_. The resulting stoichiometric model can again be represented by a stoichiometric matrix **N**, certain flux bounds [Eq. (2)], and the metabolite balancing Eq. (1).

Note also that the biomass composition **F** [and thus the stoichiometries in Eq. (5)] represent a new degree of freedom (not contained in single-species models) which we varied in the simulations in the “Results and discussion” section.

#### Objective functions

Metabolic stoichiometric models are usually underdetermined (infinite many flux vectors exist). To predict certain metabolic characteristics of an organism, linear optimizations are carried out (FBA). A frequently applied objective function in Eq. (3) is the maximization of the growth rate or biomass yield. It is reasonable to use this rationale also for community models and thus to maximize the community growth rate *µ*_*C*_:

6 $$\mathop {\text{maximize}}\limits_{\bf{r}} \;\;\;\;\mu_{C}$$

[subject to Eqs. (1) and (2) and a given community composition **F**]. However, as it turns out, while the maximum community growth rate (which we denote here by $\mu_{C}^{{^{\text{opt}} }}$) is unique, the corresponding flux distributions resulting in the optimal community growth rate are often not unique. Furthermore, many solutions might reflect unrealistic behaviors where one or more organisms must behave “altruistically” and waste energy because the maximum community growth rate is limited by another organism (see “Results and discussion” section).

We therefore follow a hierarchical optimization approach and use as a reasonable second optimization criterion that the cells not only grow with maximum growth rate but additionally also under optimal utilization of substrates, i.e., with maximum biomass yields. Since the species in the community use different substrates resulting in different maximum biomass yields (even in case of identical substrates the maximum biomass yields might be different), we need to simultaneously consider maximization of biomass yield for each organism. Our objective function for the secondary optimization step therefore contains one term for each organism and substrate, namely the product of the substrate uptake rate $r_{{{\text{S}}_{i} }}$ of substrate S_*i*_ in species *i* with the specific maximum theoretical biomass yield $\mathbf{Y}_{{X_{i} /{\text{S}}_{i} }}^{{\hbox{max} ,\mu_{C}^{\text{opt}} }}$ of species *i* on its substrate S_*i*_ at the maximum growth rate $\mu_{C}^{{^{\text{opt}} }}$ [determined in the first optimization step (6)]:

7 $$\begin{array}{*{20}c} {\mathop {\text{minimize}}\limits_\mathbf{r} \;z\; = \;r_{{{\text{S}}_{1} }} \cdot \mathbf{Y}_{{X_{1} /{\text{S}}_{1} }}^{{\hbox{max} ,\,\mu_{C}^{\text{opt}} }} + r_{{{\text{S}}_{2} }} \cdot \mathbf{Y}_{{X_{2} /{\text{S}}_{2} }}^{{\hbox{max} ,\,\,\mu_{C}^{\text{opt}} }} + \cdots + r_{{{\text{S}}_{n} }} \cdot \mathbf{Y}_{{X_{n} /{\text{S}}_{n} }}^{{\hbox{max} ,\,\,\mu_{C}^{\text{opt}} }} } \\ {s.t.} \\ {\mu_{C} = \mu_{C}^{\text{opt}} } \\ \mathbf{Nr = 0} \\ {{\it{lb}}_{j} \le r_{j} \le {\it{ub}}_{j} } \\ \mathbf{F} \\ \end{array}$$

Importantly, $\mathbf{Y}_{{X_{i} /{\text{S}}_{i} }}^{{\hbox{max} ,\,\,\mu_{{{\text{C}}}}^{opt} }}$ is determined in the single-species model at the maximum biomass yield of species *i* on its substrate S_*i*_ for the fixed (maximum) community growth rate $\mu_{C}^{{^{\text{opt}} }}$. Furthermore, if a species uses multiple substrates, one summand must be included in Eq. (7) for each substrate used. As also indicated in Eq. (7), the secondary optimization is subject to (i) the given community composition*** F***, (ii) the determined maximum community growth rate $\mu_{C}^{{^{\text{opt}} }}$, and (iii) the usual steady-state conditions and flux bounds. Consequently, the optimum *z*^opt^ resulting from the optimization of (7) is the (theoretical) minimal total biomass synthesis rate [gDW_C_/(gDW_C_ h)], we could expect for the specified conditions *if* all organisms would grow with maximum biomass yields. If the value of *z*^opt^ equals the value of the assumed community growth rate $\mu_{C}^{{^{\text{opt}} }},$ then we can conclude that all species grow with maximal biomass yields, otherwise at least one species must “waste” some substrate to keep the community in a balanced state. To quantify “how close” the community is with respect to the second optimization criterion (optimal substrate utilization (biomass yield) of all substrates/organisms involved), we calculate the OptDeg as the quotient of the maximal community growth rate $\left( {\mu_{C}^{{^{\text{opt}} }} } \right)$ and the minimal expected growth rate *z*^opt^ if the cells use their substrates optimally:

8 $${\text{OptDeg}} = \mu_{C}^{\text{opt}} /z^{\text{opt}}$$

OptDeg is smaller than one if at least one organism “wastes” its substrate. It reaches unity if the community grows optimally with respect to both growth rate and biomass yield which we consider as the most likely behavior of the species in the community (see “Results and discussion”). As described above, in several simulations, we will calculate *z*^opt^ (and OptDeg) as secondary objective based on a given optimal community growth rate $\mu_{C}^{{^{\text{opt}} }}$ (the primary objective). However, in some calculations, we will also study biomass yield optimality for any *fixed* community growth rate *µ*_*C*_. This is relevant, for example, for a continuous process with a constant dilution (and thus constant specific community growth) rate.

#### Simulations

As described in the Introduction section and as indicated in Fig. 1, we assumed that hydrogen and formate produced by *D. vulgaris* must be consumed by the methanogenic organisms (*M. maripaludis*, *M. barkeri*). Acetate accumulation, on the other hand, was allowed in the simulations. We fixed the community composition *F* for single simulations. In community simulation studies, we scanned the whole range of possible community compositions by discretizing the specific fraction *F*_*i*_ of each organism from 0 to 1 in steps of 0.01 (while ensuring that the sum of all *F*_*i*_ must be unity).

We implemented and analyzed all models with *CellNetAnalyzer*, a MATLAB package for structural and functional analysis of metabolic and signaling networks [47] which itself uses CPLEX as solver for linear optimizations. All models discussed in this work are documented (Additional file 2) and available in SBML format (Additional files 3, 4, 5, 6 and 7).

### Biomass estimation from the literature data

For comparison of simulation results with experimental data from Meyer et al. [36, 48], we needed to derive the total biomass produced in the experiments. Meyer et al. measured the cell concentrations, which we used to approximate the dry weight concentrations. *M. maripaludis* builds spherical cells with a diameter of approximately 1 µm. For *Desulfovibrio* species, we assumed a rod shape (cylinder with half spheres as ends) with a length of 2 µm and a diameter of 0.5 µm. The calculated cell volumes were 0.5 µm^3^ and 0.3 µm^3^, respectively. For approximating the dry weight per cell, we took the relation of dry weight to cell volume from Loferer-Krossbacher et al. [49]: m = 435*V^0.85^ (m is the dry weight in fg and V is the volume in µm^3^). With the calculated dry weights per cell, we transformed the cell ratio into a dry weight ratio and calculated substrate consumption and product formation rates.

## Additional files

10.1186/s13068-016-0429-x Investigation of proton translocation stoichiometries in *D. vulgaris*

10.1186/s13068-016-0429-x Documentation for all stoichiometric models used in this study.
10.1186/s13068-016-0429-x Model of *D. vulgaris* in SBML format.
10.1186/s13068-016-0429-x Model of M. barkeri in SBML format.
10.1186/s13068-016-0429-x Model of M. maripaludis in SBML format.
10.1186/s13068-016-0429-x Two-species model in SBML format.
10.1186/s13068-016-0429-x Three-species model in SBML format.

## Abbreviations

OptDeg: optimality degree
ATPmaint: ATP maintenance coefficient
FBA: flux balance analysis
