# Supplementary material for: Predicting compositions of microbial communities from stoichiometric models with applications for the biogas process
Source: Biotechnol Biofuels. 2016 Jan 22;9:17. doi: 10.1186/s13068-016-0429-x (PMC4724120; doi:10.1186/s13068-016-0429-x)
Supplement: Supplementary file 1 — 10.1186/s13068-016-0429-x Investigation of proton translocation stoichiometries in D. vulgaris [file 13068_2016_429_MOESM1_ESM.pdf]

Additional File 1

Predicting compositions of microbial communities from stoichiometric models with applications for the biogas process

Sabine Koch[1], Dirk Benndorf[2], Karen Fronk[3], Udo Reichl[1,2], Steffen Klamt[1,#]

[1]Max Planck Institute for Dynamics of Complex Technical Systems
Sandtorstr. 1, 39106 Magdeburg, Germany

[2]Otto-von-Guericke-University
Universitätsplatz 2, 39106 Magdeburg

[3]Harz University of Applied Sciences
Friedrichstrasse 57-59, 38855 Wernigerode

koch@mpi-magdeburg.mpg.de

benndorf@mpi-magdeburg.mpg.de

kfronk@hs-harz.de

ureichl@mpi-magdeburg.mpg.de

klamt@mpi-magdeburg.mpg.de

Running title: Prediction of microbial community compositions

[#]Corresponding author:
Steffen Klamt
Max Planck Institute for Dynamics of Complex Technical Systems
Sandtorstrasse 1
D-39106 Magdeburg, Germany
Phone: ++49 391 6110 480
Email: klamt@mpi-magdeburg.mpg.de

Investigation of proton translocation stoichiometries in *D. vulgaris*

Several recent studies analyzed electron transport processes relevant for growth on ethanol and other substrates (Meyer et al. 2013a; Meyer et al. 2013b; Meyer et al. 2014; Pereira et al. 2011; Price et al. 2014). Accordingly, we included reactions in the stoichiometric model of *D. vulgaris* for the ferredoxin oxidation from Coo- or Ech-hydrogenases and the Rnf complex which are present in different *Desulfovibrio* species (Meyer et al. 2014; Pereira et al. 2011; Price et al. 2014; Walker et al. 2009), and used by the organisms to create a proton gradient (Buckel and Thauer 2013; Price et al. 2014) (eq. (S1) and (S2)). Regarding the NADH oxidation, we added the electron bifurcation mechanism by the Flox/Hdr-complex that has been described by different authors (Meyer et al. 2014; Pereira et al. 2011; Walker et al. 2009). The oxidation of two NADH is coupled to the reduction of one ferredoxin and the reduction of a heterodisulfide (RS) (eq. (S3)). Electrons from lactate oxidation are transferred to a heterodisulfide (RS). For the metabolic model, we assumed that the energetically unfavorable oxidation of the heterodisulfide consumes energy and thus coupled this oxidation with the use of a proton gradient (eq. (S4)).

$$1\ Fd_{red} \leftrightarrow 1\ Fd_{ox} + 1\ H_2 + ?\ H^+(ex) \quad (S1)$$

$$1\ Fd_{red} + 1\ CO_2 \leftrightarrow 1\ Fd_{ox} + 1\ \text{formate} + ?\ H^+(ex) \quad (S2)$$

$$1\ NADH + 0.5\ Fd_{ox} + 0.5\ RS \leftrightarrow 1\ NAD^+ + 0.5\ Fd_{red} + 0.5\ RSH_2 \quad (S3)$$

$$1\ RSH_2 + ?\ H^+(ex) \leftrightarrow 1\ RS + H_2 \quad (S4)$$

The numbers of translocated protons for equations (S1), (S2) and (S4) are not clear and we therefore used experimental data to estimate them (see below).

Furthermore, we assumed that NAD^+ is the electron acceptor for ethanol oxidation to acetaldehyde and electrons are transferred from acetaldehyde to ferredoxin by acetaldehyde

oxidoreductase because the potential of this reaction is low enough to use ferredoxin as an electron acceptor (Buckel and Thauer 2013; Hensgens et al. 1995). Depending on the number of protons translocated, the two oxidation steps could yield enough energy to support growth on ethanol without sulfate reduction.

Another possibility for growth on ethanol without the need for sulfate reduction would be substrate level phosphorylation via coenzyme A linked acetaldehyde dehydrogenase which was shown to produce acetyl-CoA from acetaldehyde in *E. coli* (Shone and Fromm 1981). However, experimental data from Kremer et al. did not support this assumption (Kremer et al. 1988) and thus we did not include this pathway in the model.

Validation with experimental data

To identify the open stoichiometries of translocated protons in equations (S1)-(S4) we calculated the (maximum) biomass yields for *D. vulgaris* that follow for the different stoichiometries for growth on the substrates lactate, pyruvate and ethanol in syntrophic conditions and in combination with sulfate (Table S1). These calculated results are in the following compared with experimental data to determine the stoichiometries that fit these data best. Unfortunatley, we could not find suitable data for growth on ethanol, and biomass yields for lactate differed largely in the literature data. For growth on lactate with sulfate Cappenberg et al. calculated a biomass yield of 30.6 g/mol (Cappenberg 1975) while Okabe et al. and Okabe and Characklis reported much lower values between 2.5 g/mol and 3.7 g/mol (Okabe and Characklis 1992; Okabe et al. 1992). Due to this large difference we focused on semi-quantitative data.

Table S1: Model-derived maximum biomass yields in [gDW/mol substrate] for different organic substrates for *D. vulgaris* under syntrophic and sulfate-reducing conditions for different stoichiometries of translocated protons for ferredoxin oxidation via Ech-/Coo-hydrogenase and heterodisulfide oxidation. The shaded row represents the stoichiometry that was chosen for the following simulations

Translocated H^+ Ech/Coo (eqs. (S1) + (S2))	Translocated H^+ heterodisulfide oxidation (eq. (S4))	Lactate	Lactate + sulfate	Pyruvate	Pyruvate + sulfate	Ethanol	Ethanol + sulfate
0	0	8.45	13.64	8.45	10.72	0	5.85
0	1	4.37	10.05	8.74	11.06	0	4.02
0	2	0	6.23	9.06	11.42	0	2.08
1	0	11.75	16.34	11.75	13.61	5.87	10.89
1	1	8.08	13.08	12.12	14.01	4.04	9.34
1	2	4.17	9.63	12.52	14.44	2.09	7.70
2	0	14.58	18.69	14.58	16.14	10.94	15.29
2	1	11.26	15.71	15.02	16.58	9.38	13.97
2	2	7.74	12.57	15.47	17.05	7.74	12.57

Several authors observed that growth on pyruvate was faster and resulted in higher biomass yields compared to lactate (Bryant et al. 1977; Cooney et al. 1996; Kremer et al. 1988) The biomass yield ratio for growth on lactate to growth on pyruvate was determined to be 0.69 (Walker et al. 2009). Nagpal et al. (2000) and Kremer et al. (1988) also reported that there is only little growth on ethanol and that the biomass yields are lower than on lactate.

In the model, growth on ethanol without sulfate is only supported with at least one translocated proton during ferredoxin oxidation. The biomass yield for growth on pyruvate is only higher than the biomass yield achieved with lactate if protons are translocated for heterodisulfide oxidation. Overall, the experimental observations described above are best

represented by a stoichiometry of one translocated proton for ferredoxin oxidation (eqs. (S1), (S2)) and two protons for heterodisulfide oxidation (eq. (S4)).

Due to the high variation or lack of experimental data in literature there remains some degree of uncertainty in this part of the constructed model. Possible reasons for variation in experimental data are different culturing conditions like medium composition, pH and temperature that can influence the biomass yields.

References

Bryant MP, Campbell LL, Reddy CA, Crabill MR. 1977. Growth of desulfovibrio in lactate or ethanol media low in sulfate in association with H2-utilizing methanogenic bacteria. Appl Environ Microbiol 33(5):1162-9.

Buckel W, Thauer RK. 2013. Energy conservation via electron bifurcating ferredoxin reduction and proton/Na(+) translocating ferredoxin oxidation. Biochim Biophys Acta 1827(2):94-113.

Cappenberg TE. 1975. A study of mixed continuous cultures of sulfate-reducing and methane-producing bacteria. Microb Ecol 2(1):60-72.

Cooney MJ, Roschi E, Marison IW, Comminellis C, von Stockar U. 1996. Physiologic studies with the sulfate-reducing bacterium< i> Desulfovibrio desulfuricans</i>: Evaluation for use in a biofuel cell. Enzyme Microb Technol 18(5):358-365.

Hensgens CM, Hagen WR, Hansen TA. 1995. Purification and characterization of a benzylviologen-linked, tungsten-containing aldehyde oxidoreductase from Desulfovibrio gigas. J Bacteriol 177(21):6195-200.

Kremer DR, Nienhuiskuiper HE, Hansen TA. 1988. Ethanol Dissimilation in Desulfovibrio. Arch Microbiol 150(6):552-557.

Meyer B, Kuehl J, Deutschbauer AM, Price MN, Arkin AP, Stahl DA. 2013a. Variation among Desulfovibrio Species in Electron Transfer Systems Used for Syntrophic Growth. J Bacteriol 195(5):990-1004.

Meyer B, Kuehl JV, Deutschbauer AM, Arkin AP, Stahl DA. 2013b. Flexibility of Syntrophic Enzyme Systems in Desulfovibrio Species Ensures Their Adaptation Capability to Environmental Changes. J Bacteriol 195(21):4900-4914.

Meyer B, Kuehl JV, Price MN, Ray J, Deutschbauer AM, Arkin AP, Stahl DA. 2014. The energy-conserving electron transfer system used by Desulfovibrio alaskensis strain G20 during pyruvate fermentation involves reduction of endogenously formed fumarate and cytoplasmic and membrane-bound complexes, Hdr-Flox and Rnf. Environ Microbiol 16(11):3463-86.

Nagpal S, Chuichulcherm S, Livingston A, Peeva L. 2000. Ethanol utilization by sulfate-reducing bacteria: an experimental and modeling study. Biotechnol Bioeng 70(5):533-43.

Okabe S, Characklis WG. 1992. Effects of temperature and phosphorous concentration on microbial sulfate reduction by Desulfovibrio desulfuricans. Biotechnol Bioeng 39(10):1031-42.

Okabe S, Nielsen PH, Charcklis WG. 1992. Factors affecting microbial sulfate reduction by Desulfovibrio desulfuricans in continuous culture: limiting nutrients and sulfide concentration. Biotechnol Bioeng 40(6):725-34.

Pereira IA, Ramos AR, Grein F, Marques MC, da Silva SM, Venceslau SS. 2011. A comparative genomic analysis of energy metabolism in sulfate reducing bacteria and archaea. Front Microbiol 2:69.

Price MN, Ray J, Wetmore KM, Kuehl JV, Bauer S, Deutschbauer AM, Arkin AP. 2014. The genetic basis of energy conservation in the sulfate-reducing bacterium Desulfovibrio alaskensis G20. Front Microbiol 5:577.

Shone CC, Fromm HJ. 1981. Steady-state and pre-steady-state kinetics of coenzyme A linked aldehyde dehydrogenase from Escherichia coli. Biochemistry 20(26):7494-501.

Walker CB, He Z, Yang ZK, Ringbauer JA, Jr., He Q, Zhou J, Voordouw G, Wall JD, Arkin AP, Hazen TC and others. 2009. The electron transfer system of syntrophically grown Desulfovibrio vulgaris. J Bacteriol 191(18):5793-801.
